# Artificial metabzyme‐driven metabolic reprogramming and precision oncology

**DOI:** 10.1002/ctm2.70215

**Published:** 2025-01-30

**Authors:** Xi Hu, Daishun Ling

**Affiliations:** ^1^ School of Pharmacy Anhui University of Chinese Medicine Hefei China; ^2^ Institute of Pharmaceutics Anhui Academy of Chinese Medicine Hefei China; ^3^ Anhui Province Key Laboratory of Pharmaceutical Preparation Technology and Application Anhui University of Chinese Medicine Hefei China; ^4^ Frontiers Science Center for Transformative Molecules School of Chemistry and Chemical Engineering National Center for Translational Medicine, National Engineering Research Center of Advanced Magnetic Resonance Technologies for Diagnosis and Therapy Shanghai Jiao Tong University Shanghai China; ^5^ WLA Laboratories Shanghai China

1

Abnormal metabolism is a biological hallmark of cancer and represents critical targets for therapeutic intervention, as it unveils potential vulnerabilities for treatment.^1^ To sustain continuous proliferation and metastasis, tumour cells undergo several metabolic adaptations to cope with the nutrient‐deficient microenvironment. Recent advancements have demonstrated the successful translation of identified metabolic dysregulations in cancer cells into FDA‐approved metabolic inhibitors. Currently, several metabolic regulators are being developed or are undergoing clinical trials for the treatment of various cancers, such as nucleotide synthesis inhibitors (e.g. aminopterin, methotrexate and pemetrexed), indoleamine 2,3‐dioxygenase 1 inhibitors (e.g. linrodostat and KHK2455), isocitrate dehydrogenases inhibitors (e.g. ivosidenib and enasidenib), glutaminase inhibitors (e.g. telaglenastat and telaglenastat), lactate efflux inhibitors (e.g. AZD3965), tyrosine mimetics (e.g. racemetyrosine), and so on.^2^, ^3^ However, despite significant advancements in the development of drugs targeting cancer genomic alterations and the tumour microenvironment, the progress in targeting cancer metabolism—particularly non‐nucleotide metabolism—remains in its nascent stages. A major challenge in targeting cancer metabolism for therapy lies in achieving effective antitumour effects while minimizing toxicity to normal cells, as many metabolic pathways essential for tumour cell survival are also shared by normal cells, resulting in a narrow therapeutic window and potential for significant toxicity.^4^

Xanthine oxidoreductase (XOR), a key enzyme in purine catabolism containing redox‐active molybdenum (Mo) and iron (Fe) centres, catalyses the oxidation of hypoxanthine to xanthine and xanthine to uric acid (UA).^5^ Its expression and activity are significantly reduced in tumour tissues from liver, breast, gastrointestinal, colorectal, ovarian and non‐small cell lung cancers, with low XOR levels strongly associated with poor prognosis and recurrence.^6^, ^7^ Moreover, the documented immunosuppressive properties of certain xanthine derivatives^8^ and the notable role of UA in enhancing anti‐tumour immunity^9^ underscore the pivotal relevance of XOR in cancer research, suggesting its potential as both a therapeutic target and a mediator of immune responses. Leveraging this insight, we engineered FeMoO_4_ nanocatalysts, an artificial metabzyme graced with Fe^2+^ and tetrahedral Mo^4+^ active centres, to seamlessly simulate XOR's catalytic essence.^10^ Upon entering tumour cells with low XOR levels and elevated xanthine substrates, the FeMoO_4_ metabzyme efficiently catalyses the conversion of xanthine into excess UA. Interestingly, UA metabolite, in turn, triggers macrophages to release proinflammatory cytokines, such as interleukin‐1β (IL‐1β), promoting the polarization of immunostimulatory M1 macrophages and activating other immune cells, including dendritic cells (DCs) and T cells. Our design paves the way for the development of advanced artificial metabzymes, enabling tumour cells to undergo metabolic reprogramming and then autonomously initiate direct crosstalk with immune cells, thereby advancing tumour‐cell‐specific metabolic therapy (Figure 1).

**FIGURE 1:**
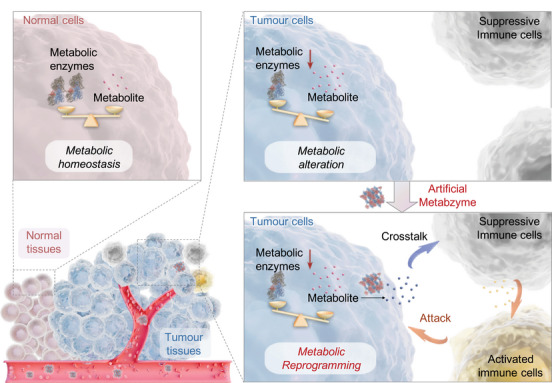
Schematic illustration of the artificial metabzyme for tumour‐cell‐specific metabolic therapy. In stark contrast to normal cells, tumour cells exhibit distinct alterations in natural enzymes (e.g. XOR), resulting in the accumulation of specific metabolites (e.g. xanthine). On entering the tumour cells, the artificial metabzyme efficiently catalyzes the conversion of these abundant metabolites into other small‐molecule metabolites (e.g. UA), which, notably, further might facilitate tumour‐immune cell crosstalk, activating immune cells and thereby enabling tumour‐cell‐specific metabolic therapy.

The crosstalk between tumour cells and immune cells plays a pivotal role in both tumour progression and the response to cancer therapies.^11^ Recent advances in cancer immunotherapy have focused on modulating this immune‐tumour crosstalk, with strategies like immune checkpoint inhibitors, cancer vaccines, and cell‐based therapies.^12^ However, tumours often develop mechanisms to evade immune surveillance, such as compensatory upregulation of alternative immune checkpoints (e.g. T‐cell immunolgobulin and mucin domain containing protein‐3 [TIM‐3], lymphocyte‐activation geng‐3 [LAG‐3] and V‐domain Ig suppressor of T cell activation [VISTA]), tumour antigen loss, metabolic reprogramming, and heterogeneous tumour evolution, thereby complicating the efficacy of immunotherapies.^13^, ^14^ Indeed, tumour cell metabolites play a crucial role as signalling molecules that influence the interaction between tumour cells and immune cells.^15^ More importantly, tumour‐derived metabolites may function as “danger signals,” triggering immune responses that can inhibit tumour progression. For instance, UA has been reported to activate macrophages to excrete proinflammatory cytokine IL‐1β through the UA‐NLRP3‐IL‐1β signalling pathway, where IL‐1β, in turn, promotes the M1 macrophage polarization and activates other immune cells (e.g. DCs and T cells), thereby enhancing anti‐tumour immunity.^9^, ^16^ In our study, the XOR‐mimicking FeMoO_4_ metabzyme reprograms tumour cell xanthine metabolism, with the resulting UA metabolite facilitating metabolic crosstalk with neighbouring immune cells and enabling a highly efficient cancer metabolic therapy specifically targeting tumour tissues.^10^ Therefore, reprogramming the metabolic landscape of tumour cells offers the potential to redirect the immune response toward a more anti‐tumour phenotype, enhancing the efficacy of cancer immunotherapies, overcoming immune evasion mechanisms, and simultaneously minimizing off‐target side effects.

Collectively, our findings highlight xanthine metabolism as a promising therapeutic target and UA as a metabolic immune checkpoint agonist specifically directed at tumour cells, thereby opening new avenues for metabolism‐driven precision oncology. However, further research is necessary to investigate the complex metabolic pathways specific to tumour cells and identify potential targets for metabolic and immune regulation—an area that should be prioritized by both clinicians and researchers with the aim of discovering novel and rational combinations of clinical drugs. Moreover, our ‘metabzyme’ concept could pave the way for the emerging field of ‘artificial metabolic enzyme replacement therapy’. Additional metabolic enzyme targets warrant exploration as potential therapeutic targets for metabolic diseases, including cancer, diabetes, and cardiovascular disorders, thereby establishing the physiological foundation for the clinical design and development of ‘metabzymes’.

## AUTHOR CONTRIBUTIONS

Xi Hu wrote the manuscript, and Daishun Ling revised the manuscript. All the authors reviewed and approved the final version of the manuscript.

## CONFLICT OF INTEREST STATEMENT

The authors declare no conflict of interest.

## Data Availability

Data sharing is not applicable to this article as no datasets were generated or analysed during the current study.
